# Chinese International Students’ Satisfaction with University Mental Health Counselling: A Mixed-Methods Study on the Impacts of Language Barriers, Cultural Differences and the Working Alliance

**DOI:** 10.3390/healthcare14183010

**Published:** 2026-09-14

**Authors:** Xiang Meng, Nicholas Lee

**Affiliations:** Department for Education Studies, The University of Warwick, Coventry CV4 7AL, UK; xiang.meng.1@warwick.ac.uk

**Keywords:** university counseling services, language and cultural barriers, mixed-methods study, counselling satisfaction

## Abstract

**Background**: Chinese international students may experience language and cultural challenges when using university counselling services. This study examined the associations between perceived language barriers, cultural differences, and counselling satisfaction and explored students’ counselling experiences using a mixed-methods approach. **Methods**: An explanatory sequential mixed-methods design was used. A total of 125 Chinese international students with previous university counselling experience completed an online questionnaire assessing counselling satisfaction, perceived language barriers, and cultural differences. Descriptive statistical analysis, group comparisons, correlation analysis, and multiple regression were conducted. Subsequently, 10 participants completed semi-structured interviews. Interview data were analyzed using reflexive thematic analysis, and qualitative findings were integrated with the quantitative results. **Results**: Perceived language barriers and cultural differences were negatively associated with counselling satisfaction in bivariate analyses. In the regression model, cultural differences showed the strongest negative association with counselling satisfaction, whereas language barriers were no longer statistically significant. The model explained 18.2% of the variance in counselling satisfaction. Qualitative findings indicated that second-language use could create cognitive and communicative difficulties but could also provide emotional distance that facilitated disclosure. Participants also emphasized empathy, cultural responsiveness, trust, goal alignment, and therapeutic collaboration. **Conclusions**: Cultural differences may be particularly important in understanding counselling satisfaction among Chinese international students, while the role of language appears complex rather than uniformly negative. Alliance-related experiences may help students navigate linguistic and cultural difficulties. Further research should use validated measures of working alliance and language and cultural barriers.

## 1. Introduction

University students’ mental health is increasingly recognized as a public health priority, with students typically entering higher education during a developmental stage that seems to carry a heightened risk for the onset of mental health issues [1,2]. Within this broader context, international students are a particularly vulnerable subgroup who face challenges with counselling [3].

According to the UNESCO Institute for Statistics, more than 6.4 million students were enrolled in tertiary education outside their home countries worldwide in 2023, and Chinese students continue to represent one of the largest international student populations globally. Despite increasing awareness of student mental health, international students consistently report lower utilization of university counselling services than domestic students. Among Chinese international students, concerns about language barriers, cultural stigma, and limited trust in mental health professionals remain among the most frequently reported barriers to help-seeking. These trends highlight the growing need to better understand factors influencing counselling satisfaction in culturally diverse university settings.

Among the diverse international student population, Chinese students constitute one of the largest groups within the global population of international students. It has been illustrated that there is an increased level of anxiety, depression, and loneliness among Chinese international students [4], which correlates with the increased need for campus mental services. Despite the considerable need for psychological support, Chinese international students often encounter distinctive challenges when seeking counselling services [5,6].

Existing studies have consistently identified language and cultural differences as major determinants of Chinese international students’ counselling experiences [7,8]. The L2 cognitive load hypothesis posits that processing and communicating in a non-native language increases cognitive demands, leading to reduced emotional depth, expressiveness, and spontaneity. In contrast, the affective distance hypothesis suggests that using a second language can create psychological distancing from distressing emotions [9]. According to the study of [10], linguistic distancing may improve therapeutic openness and honesty, which may strengthen the working alliance. Cultural differences represent another important dimension of counselling experiences. In many Asian communities, professional counselling may be associated with stigma or concerns about social judgement, which can reduce willingness to seek psychological support [11,12]. Cultural incongruence may also influence how clients interpret counsellors’ communication styles, therapeutic expectations, and expressions of empathy. Previous research has shown that perceived cultural mismatch can negatively shape Asian clients’ evaluations of counselling, highlighting the importance of culturally responsive practice [13]. In contrast, cultural humility emphasizes counsellors’ openness, self-reflection, adaptability, and willingness to learn from clients’ cultural perspectives [14]. These contrasting possibilities suggest that culture should not be understood solely as a fixed barrier; rather, its influence may depend partly on the degree of cultural congruence and counsellor responsiveness within the counselling relationship.

Previous studies [15] have treated language-related and cultural factors as two distinct but potentially interacting variables influencing Chinese international students’ counselling experiences. The existing literature suggests that both can present notable barriers to engagement and satisfaction [16]. Although previous studies have demonstrated that both language barriers and cultural differences influence international students’ counselling experiences, several important gaps remain. First, existing research has generally examined language and culture as independent predictors, with limited attention to how these factors interact in shaping counselling satisfaction. Second, although the working alliance has consistently been identified as a key determinant of psychotherapy outcomes, relatively few studies have explored how alliance-related processes may help explain international students’ counselling experiences in cross-cultural contexts. Third, most previous studies have relied exclusively on quantitative surveys or qualitative interviews, while comparatively few have adopted an integrated mixed-methods approach capable of explaining statistical findings through participants’ lived experiences. These limitations motivate the present study.

Therefore, this study adopted an explanatory sequential mixed-methods design to investigate how perceived language barriers and cultural differences are associated with counselling satisfaction among Chinese international students. Quantitative findings were subsequently elaborated through qualitative interviews to better understand the experiences underlying statistical patterns. Rather than testing the working alliance as a mediator, the qualitative analysis explored how alliance-related experiences, including goal agreement, emotional bonding, and therapeutic tasks, may help explain counselling satisfaction across diverse counselling contexts.

The remainder of this paper is structured as follows. Section 2 describes the materials and methods, including the study design, participants, measures, and data analysis procedures. Section 3 presents the quantitative results, followed by the qualitative findings and their integration through a joint display. Section 4 discusses the principal findings in relation to the existing literature, theoretical mechanisms, and practical implications for university counselling services. Finally, Section 5 concludes the study with a summary of contributions, acknowledges the limitations, and outlines directions for future research.

The study integrates quantitative associations between perceived language and cultural barriers and counselling satisfaction with qualitative accounts explaining how language, cultural congruence, and alliance-related experiences are experienced during counselling.

## 2. Materials and Methods

Ethical approval for the study was granted on 17 June 2025 by Education Studies under the authority of the Humanities and Social Science Research Ethics Committee (HSSREC) at the University of Warwick. Two faculty members of the Education Studies Department independently conducted the ethical review of the research proposal. As this was a student research project, no separate ethical approval or reference number was issued. Only after securing this approval did the actual data collection process begin. All participants were provided with a comprehensive information leaflet explaining the study’s purpose, procedures, potential risks and benefits, confidentiality measures, and data handling protocols. Written informed consent was obtained from all participants prior to data collection, and participants were informed of their right to withdraw from the study at any time. All questionnaire data were collected anonymously, and identifiable information from the interview data was removed during transcription to protect participants’ privacy.

### 2.1. Study Design

This study adopted an explanatory sequential mixed-methods design. Quantitative data were collected first to examine the associations among perceived language barriers, cultural differences, and counselling satisfaction. Qualitative interviews were subsequently conducted to explain and elaborate on the quantitative findings, particularly the unexpected non-significant association between language barriers and counselling satisfaction after controlling for cultural differences. Integration occurred during interpretation through comparison of quantitative statistical results with qualitative themes.

**Figure 1:**
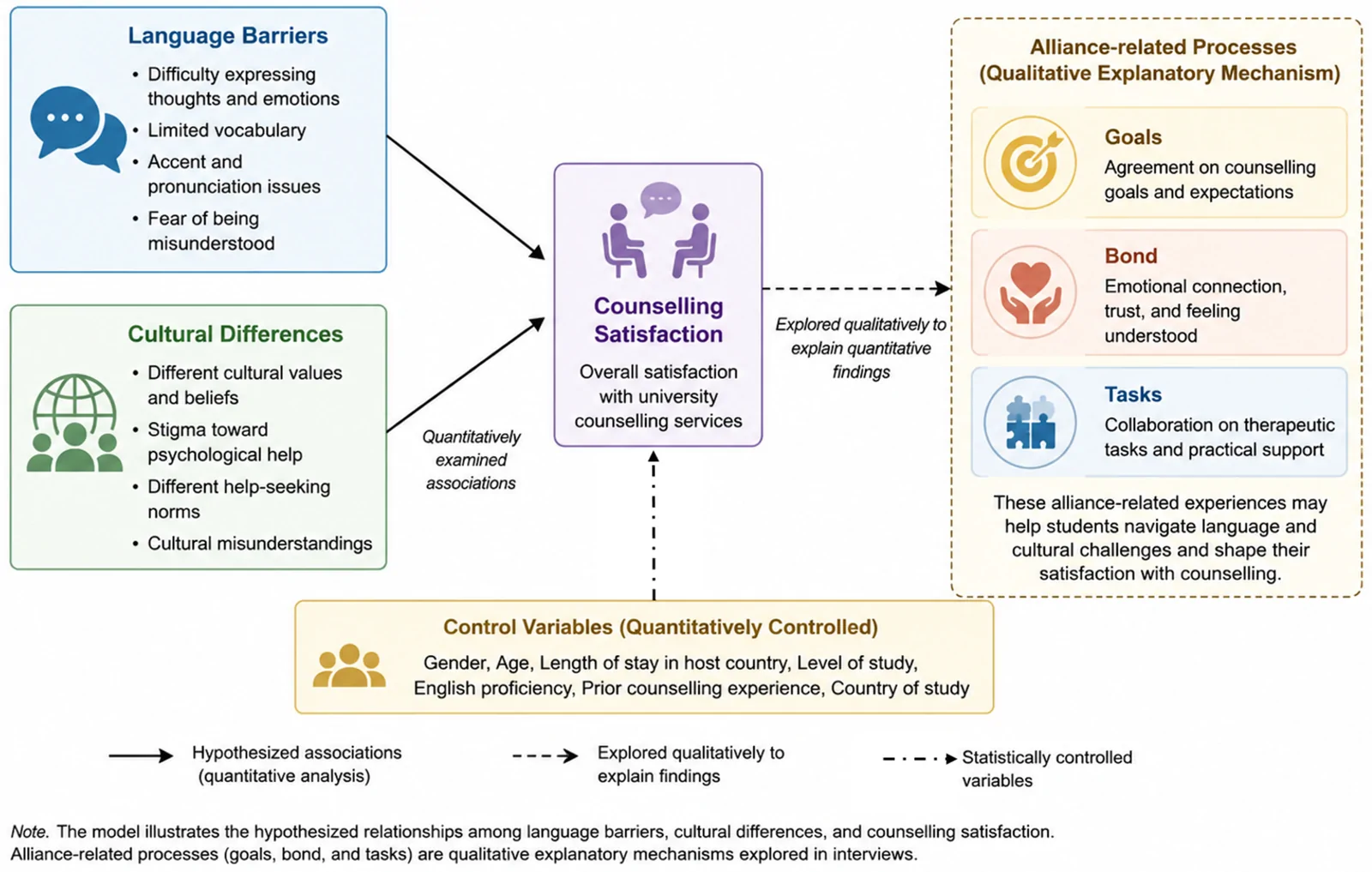
Conceptual framework of the present study.

Figure 1 presents the conceptual framework guiding this study. Based on the previous literature, language barriers and cultural differences were expected to be associated with counselling satisfaction. The qualitative component further explored how alliance-related experiences—including goal agreement, emotional bonding, and collaborative therapeutic tasks—may help explain these relationships.

Rather than viewing these theoretical perspectives independently, the present study considers them as complementary lenses for understanding counselling satisfaction among Chinese international students. Acculturation theory explains how adaptation to a new linguistic and cultural environment may shape counselling experiences. Cultural humility emphasizes counsellors’ openness, self-reflection, and responsiveness to clients’ cultural identities, which may reduce perceived cultural distance during counselling. Finally, the Working Alliance Model provides a conceptual lens for understanding how collaborative therapeutic relationships—including shared goals, emotional bonds, and agreed therapeutic tasks—may help explain why some students report positive counselling experiences despite language or cultural challenges. Importantly, in the present study, the working alliance is treated as a qualitative explanatory framework rather than as a quantitatively tested mediator or moderator.

### 2.2. Quantitative Methodology

The total number of respondents to the questionnaire, which was completed online, was 125. Snowball sampling was used to recruit the participants through a social network. A non-probability snowball sampling strategy was adopted because Chinese international students who had previously received university counselling constitute a relatively difficult-to-access population. Initial participants were invited through the researcher’s academic network and Red Note (Xiaohongshu), after which they were encouraged to share the survey with other eligible students. Although snowball sampling may increase selection bias and reduce representativeness, it has been widely recommended for recruiting hidden or dispersed international student populations.

Red Note (Xiaohongshu) was selected because it has become one of the most widely used online social platforms among Chinese international students. Compared with general social media platforms, Red Note contains numerous overseas student communities, where users actively discuss university life, mental health, and counselling experiences. Therefore, it provided an efficient platform for reaching the target population. Recruitment information was also shared through the researcher’s personal academic and social networks. No other online recruitment platforms were used.

An a priori power analysis was conducted using G*Power 3.1 for multiple linear regression. Assuming a medium effect size (f^2^ = 0.15), α = 0.05, statistical power = 0.80, and two primary predictors, the minimum required sample size was 107 participants. Therefore, the final sample of 125 participants exceeded the recommended sample size and was considered adequate for the planned statistical analyses.

### 2.3. Qualitative Methodology

The total number of respondents to interviews was 10; interviews were performed with the use of an online meeting platform (Microsoft Teams). From the sample, ten participants were selected for semi-structured interviews, to be conducted on a voluntary basis. The interviewees were not randomly selected. They were self-selected by indicating in the final question of the questionnaire their willingness to participate in a follow-up interview. These interview participants were studying in various countries, including the United Kingdom, Canada, Australia, and Germany, providing a diverse institutional and cultural background through which they had experienced counselling services. Although this sample was not chosen based on a statistical power test or saturation approach, it was selected to capture a diverse range of significant views within the target population.

Ten participants were purposively selected from survey respondents who indicated willingness to participate in follow-up interviews. Selection sought to maximize variation in gender, country of study, counselling satisfaction, and language proficiency. Because the purpose of the qualitative phase was explanatory rather than theory generation, thematic sufficiency rather than formal data saturation guided sample size. Previous mixed-methods studies have similarly demonstrated that approximately 8–15 interviews are sufficient to explain quantitative findings when participants possess rich experience relevant to the research question.

### 2.4. Tools

#### 2.4.1. Questionnaire

The questionnaire consisted of three sections. The first collected demographic information, including age, gender, host country, duration of study abroad, English proficiency, and previous counselling experience. The second consisted of an adapted version of the Satisfaction with Therapy and Therapist Scale—Revised (STTS-R). The third included six self-developed items measuring perceived language barriers and cultural differences.

The self-developed items were developed following an extensive review of the previous literature on international student counselling and were refined through expert consultation and pilot testing involving ten Chinese international students.

The quantitative tool used in this study was an online questionnaire created using Qualtrics. It consisted of 29 items and took approximately three minutes to complete. The final version is provided in Appendix A. It included three main sections: demographic information, satisfaction with counselling services, and perceptions of language and cultural barriers. Demographic questions were used to verify participants’ eligibility, including age, nationality, language background, length of stay in the host country (less than 6 months, 6 to 12 months, more than 1 year), and prior use of university counselling services.

The Satisfaction With Therapy and Therapist Scale—Revised (STTS-R) was adapted for this study. The Outcome Variable subscale was removed; some item orders were modified based on pilot test feedback to better match participants’ thought processes; and one item, “I would recommend the program to a friend”, was changed to “I would recommend the service to other international students” to fit the study population and research questions. At the start of this section, an additional item asked how many times the participant had attended counselling, which served as a validity check for prior counselling experience. The STTS-R items were measured on a five-point Likert scale (1 = strongly disagree, 2 = disagree, 3 = neutral, 4 = agree, 5 = strongly agree). Example items include:

1. I am satisfied with the quality of the therapy I received.
2. I am now able to deal more effectively with my problems.
3. The therapist seemed to understand what I was thinking and feeling.

Six self-developed items were included to assess participants’ perceptions of language and cultural barriers in counselling. Three items addressed language-related factors, and three addressed cultural background factors. These items were also measured on a five-point Likert scale (1 = strongly disagree, 2 = disagree, 3 = neutral, 4 = agree, 5 = strongly agree). The items were:

1. Speaking in a second language made it hard to express my feelings in counseling.
2. I was worried the counselor might misunderstand me because of language.
3. I sometimes needed to translate mental-health terms mentally before answering.
4. Cultural differences (values, communication style) made counseling less comfortable.
5. I felt the counselor sometimes compared my behaviour to that of local (majority-culture) students.
6. The counselor did not show understanding of the historical and social context affecting people from my culture.

#### 2.4.2. Semi-Structured Interviews

The qualitative component consisted of semi-structured interviews conducted via Microsoft Teams. Each interview lasted between 15 and 25 min. The interview guide included open-ended questions covering four main areas:

1. Experiences of satisfaction with counselling services.
2. Language barriers encountered during counselling (for example, “How easy or difficult was it to express your thoughts and feelings in a second language? Can you give an example?”).
3. Cultural barriers in counselling (for example, “How much did your cultural background influence your comfort in counseling? Can you give an example?”).
4. Suggestions for improving access to services.
5. Alliance-related experiences during counselling, including goal agreement, emotional bonding, trust, and collaboration on therapeutic tasks (for example, “Did you feel that you and the counsellor shared a clear understanding of what you wanted to achieve in counselling?”, “Did you feel understood and accepted by the counsellor?”, and “How did you and the counsellor work together on strategies or tasks during the counselling sessions?”).

These questions were included to explore alliance-related experiences as a qualitative explanatory context rather than to measure the working alliance quantitatively or test it as a mediator or moderator.

Since a semi-structured format was employed, the questions were asked in a flexible way, with additional questions to discuss some experiences and examples in more detail according to the answers of the participants. All interviews were conducted in Chinese, as preferred by participants. The interview guide was translated by the researcher and refined through pilot testing to improve accuracy and ease of understanding. The final version is provided in Appendix B.

All the interviews were audio-taped and the spoken words transcribed verbatim, with the researcher reviewing transcripts carefully and correcting any errors. Inductive thematic analysis was done, whereby the themes were identified directly in the data, thereby improving the credibility and authenticity of the results. Additionally, reflexive journaling was employed to record personal assumptions, emotional responses and decisions regarding the data coding and interpretation. The practice favoured openness and assisted in the effort to make sure that the analysis was based on stories of participants, not researcher expectations.

Internal consistency for the language barrier subscale was acceptable (Cronbach’s α = 0.96), while the cultural difference subscale demonstrated satisfactory reliability (Cronbach’s α = 0.97).

In order to increase accessibility, the researcher, as a fluent bilingual speaker, translated all the questionnaires into simplified Chinese. Although there was no formal back-translation, translation was revised after the pilot with a view to semantic fidelity, clarity, and cultural relevancy.

### 2.5. Data Analysis

#### 2.5.1. Quantitative Data Analysis

Quantitative data analysis was conducted using SPSS 26.0. Descriptive statistics were first used to summarize participants’ demographic characteristics, satisfaction scores, perceived language barrier scores, and perceived cultural barrier scores. Inferential statistics were then applied to examine group differences and relationships among variables. Independent-samples *t*-tests were used to compare scores for each dimension (satisfaction, language barriers, cultural barriers) between two-group variables, such as gender. Because the language barrier and cultural difference items were newly developed exploratory measures consisting of three items per construct, no exploratory or confirmatory factor analysis was conducted in the present study. Their preliminary reliability was instead evaluated using Cronbach’s alpha, and their construct validity remains to be established in future research using larger independent samples. Prior to regression analysis, assumptions including normality of residuals, homoscedasticity, multicollinearity, and independence of errors were examined. Variance Inflation Factors (VIFs) were below commonly accepted thresholds, indicating no evidence of problematic multicollinearity.

In the case of checking for associations between key continuous variables, Pearson correlation analysis was employed to assess relationships that existed between satisfaction scores, language barrier scores, and cultural barrier scores. Subsequently, multiple linear regression was used to examine the unique associations of perceived language barriers and cultural differences with counselling satisfaction. Multiple linear regression was subsequently conducted with counselling satisfaction as the dependent variable and perceived language barriers and cultural differences as the primary predictors. Demographic variables were examined separately through group comparisons but were not entered as covariates in the final regression model. This specification was chosen to focus on the two theoretically central predictors examined in the present study. The absence of demographic covariates is acknowledged as a limitation of the regression analysis.

#### 2.5.2. Qualitative Data Analysis

Interviews were analyzed following Braun and Clarke’s six-step thematic analysis. Initial codes were generated independently from repeated reading of transcripts. Similar codes were subsequently grouped into candidate themes. Themes were reviewed, refined, and discussed with the research supervisor until conceptual agreement was reached. Reflexive memo writing was maintained throughout analysis to enhance transparency and reduce researcher bias.

We coded the transcripts of the interviews line by line, and codes and themes emerged inductively. Even though the initial coding process was data-driven, the corresponding theories of language barriers, acculturation, and cultural competence were referred to in the interpretation stage in order to refine and contextualize the analysis. Manual coding allows for close engagement with the data, enabling the researcher to detect subtle linguistic and cultural nuances. To ensure transparency and analytical rigour, the researcher engaged in iterative code-review cycles, refining codes and themes through multiple passes. Reflexive journaling was also employed in recording coding decisions, analytical thoughts, and positionality of the researcher. The dissertation supervisor could access selected transcripts and coding records to be viewed in order to have an independent critical evaluation of the study.

During interpretation, acculturation theory was used to contextualize participants’ experiences of adapting to the linguistic and social environment of the host country, while cultural competence and cultural humility perspectives were used to interpret accounts concerning counsellor responsiveness, cultural congruence, and perceived misunderstanding. These theoretical perspectives were used as interpretive lenses after inductive themes had been identified rather than as predetermined coding categories.

Finally, the findings derived from the quantitative and qualitative analyses were compared and integrated. This process involved examining how quantitative results, such as satisfaction scores and reported barrier intensities, aligned with or differed from qualitative themes, such as communication difficulties and cultural mismatches. The aim was to use insights from each dataset to complement and cross-validate the other, supporting a cohesive and holistic understanding of participants’ experiences.

Trustworthiness was enhanced through prolonged engagement with the data, reflexive journaling, regular supervisory discussions, and maintenance of an audit trail documenting analytical decisions.

### 2.6. Integration of Mixed Methods

**Figure 2:**
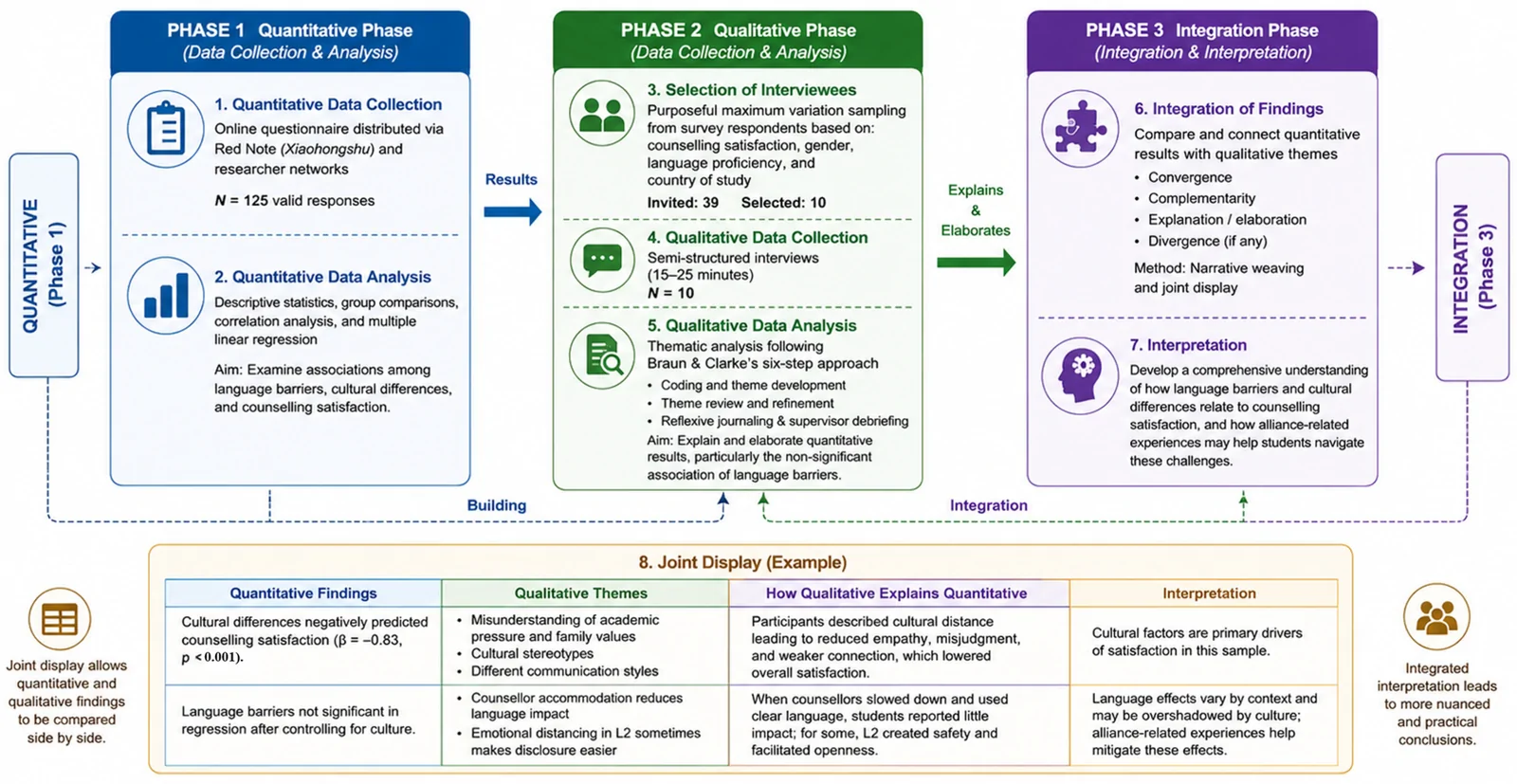
Explanatory sequential mixed-methods design of the present study.

Figure 2 illustrates the explanatory sequential mixed-methods design adopted in the present study. In Phase 1, quantitative data were collected through an online questionnaire administered to Chinese international students who had previously used university counselling services. Descriptive statistical analysis, group comparisons, correlation analyses, and multiple linear regression were conducted to identify the relationships among perceived language barriers, cultural differences, and counselling satisfaction. In Phase 2, a purposive sample of participants was selected from survey respondents to participate in semi-structured interviews. The qualitative phase was designed to provide a deeper understanding of the quantitative findings, particularly to explain why perceived language barriers were no longer statistically significant after cultural differences were included in the regression model. Finally, in Phase 3, quantitative and qualitative findings were integrated through narrative comparison and joint interpretation, allowing statistical associations to be interpreted alongside participants’ lived experiences.

Integration occurred during interpretation using a narrative weaving strategy and a joint display. Quantitative findings were first summarized statistically and then compared with qualitative themes to identify areas of convergence, complementarity, and explanation. Particular attention was given to explaining why perceived language barriers became statistically non-significant after cultural differences were included in the regression model. Rather than testing causal mechanisms, the qualitative findings were used to provide contextual explanations for quantitative associations.

Several methodological considerations should be acknowledged. First, snowball sampling may have introduced self-selection bias and limited representativeness. Second, the newly developed language and cultural difference items underwent pilot testing but were not subjected to exploratory or confirmatory factor analysis. Third, back-translation was not performed during questionnaire translation. Finally, the qualitative phase emphasized explanatory depth rather than theoretical saturation.

Because the analysis followed a reflexive thematic approach conducted by the primary researcher, formal inter-coder reliability statistics were not calculated. Analytical credibility was instead supported through iterative code review, reflexive journaling, supervisory discussion, and maintenance of an audit trail.

## 3. Results

Results are presented in three stages. First, quantitative findings are reported to examine the associations among perceived language barriers, cultural differences, and counselling satisfaction. Second, qualitative themes derived from semi-structured interviews are presented to provide a deeper understanding of students’ counselling experiences. Finally, findings from both phases are integrated to identify areas of convergence, complementarity, and explanation.

### 3.1. Quantitative Results

This research administered an online survey to a sample of Chinese international students with diverse ages, genders and durations of stay abroad. A total of 129 questionnaires were collected, of which 125 were deemed valid after excluding incomplete responses, yielding an effective response rate of 96.90%. The final sample comprised 125 valid responses, covering a range of demographic characteristics, such as gender, age, and duration of study abroad, as shown in Table 1. As shown in Table 1, participants aged 18–25 years accounted for the largest proportion (61.6%), followed by those aged 26–30 years (36.0%), while those aged 31 years and above represented only 2.4%. There was a significantly higher proportion of female participants than male participants (80.0% vs. 20.0%). Concerning the duration of stay abroad, the largest percentage was 6–12 months (46.4), followed by over 1 year (42.4), and then less than 6 months (11.2). The number of those who had reported proficient or native-level fluency in the university’s main language totaled nearly half of the sample (48.0%), and those who had reported very limited proficiency were only 0.8%.

#### 3.1.1. Current Status and Differences in Students’ Counselling Satisfaction

1. Counselling Satisfaction Scores

Table 2 presents the counselling satisfaction scores. The highest score was 60 and the lowest was 18, with a mean score of 44.53 (SD = 11.18), considered a moderate mean score out of the total possible range of the scale. The standard deviation indicates the existence of significant differences between different individuals.

2. Differences in Counselling Satisfaction

To compare experiences in relation to counselling satisfaction according to gender, an independent-samples *t*-test was conducted. The results revealed that males (M = 46.72, SD = 10.50) showed no significant difference from females (M = 43.98, SD = 11.33) (t = −1.096, *p* > 0.05), with a small effective difference (Cohen’s d = 0.25). This suggests that gender had a minimal impact on counselling satisfaction (Table 3).

A one-way ANOVA was used to analyze the differences in satisfaction with counselling in terms of age, the duration of study abroad, and the level of proficiency in the university’s main language. The only significant difference observed was in the case of age (F = 5.317, *p* < 0.01), with no significant differences in the other variables. Post hoc comparison showed that students between the ages 18 and 25 were more satisfied than their counterparts between 26 and 30, who were in turn more satisfied than those aged 31 and above (Table 4).

#### 3.1.2. Current Status and Differences in Students’ Perceived Language Barriers

1. Language Barrier Scores

Table 5 presents the scores for language barriers. Scores ranged from 3 to 15, with a mean of 12.57 (SD = 2.20), indicating a moderately high level of perceived language barriers.

2. Differences in Language Barriers

Independent-samples *t*-tests were used to test gender-based variations in language barriers, with gender as the grouping factor. The findings revealed no significant difference between males (M = 12.04, SD = 2.32) and females (M = 12.70, SD = 2.17) (t = 1.343, *p* > 0.05). The effect size, Cohen’s d = 0.29, falls into the small range. The results are shown in Table 6.

As shown in Table 7, to examine differences in language barriers based on age, duration of study abroad, and the level of proficiency in the university’s main language, one-way ANOVA tests were conducted. The results indicated no significant differences across age or duration of study abroad, but significant differences were found in primary language proficiency (F = 4.235, *p* < 0.01). However, due to two groups having sample sizes of no more than one, post hoc tests could not be conducted.

#### 3.1.3. Current Status and Differences in Students’ Perceived Cultural Differences

1. Cultural Difference Scores

The cultural difference scores of international students are presented in Table 8. As shown in Table 8, the scores ranged from 4 to 15, with a mean of 10.92 (SD = 2.89), which lies in the middle of the possible range of the scale, indicating certain individual differences in perceived cultural differences among participants.

2. Differences in Cultural Differences

To examine gender differences in cultural differences, independent-samples *t*-tests were conducted with gender as the grouping variable. The results showed no significant difference between males (M = 11.28, SD = 2.46) and females (M = 10.83, SD = 2.99) (t = –0.695, *p* > 0.05). The effect size, Cohen’s d = 0.16, falls into the small range. The results are shown in Table 9.

To examine differences in cultural differences based on age, duration of study abroad, and primary language proficiency, one-way ANOVA tests were conducted. The results indicated no significant differences across all three variables. Details are shown in Table 10.

#### 3.1.4. Correlation Analysis Among Counselling Satisfaction, Language Barriers, and Cultural Differences

**Figure 3:**
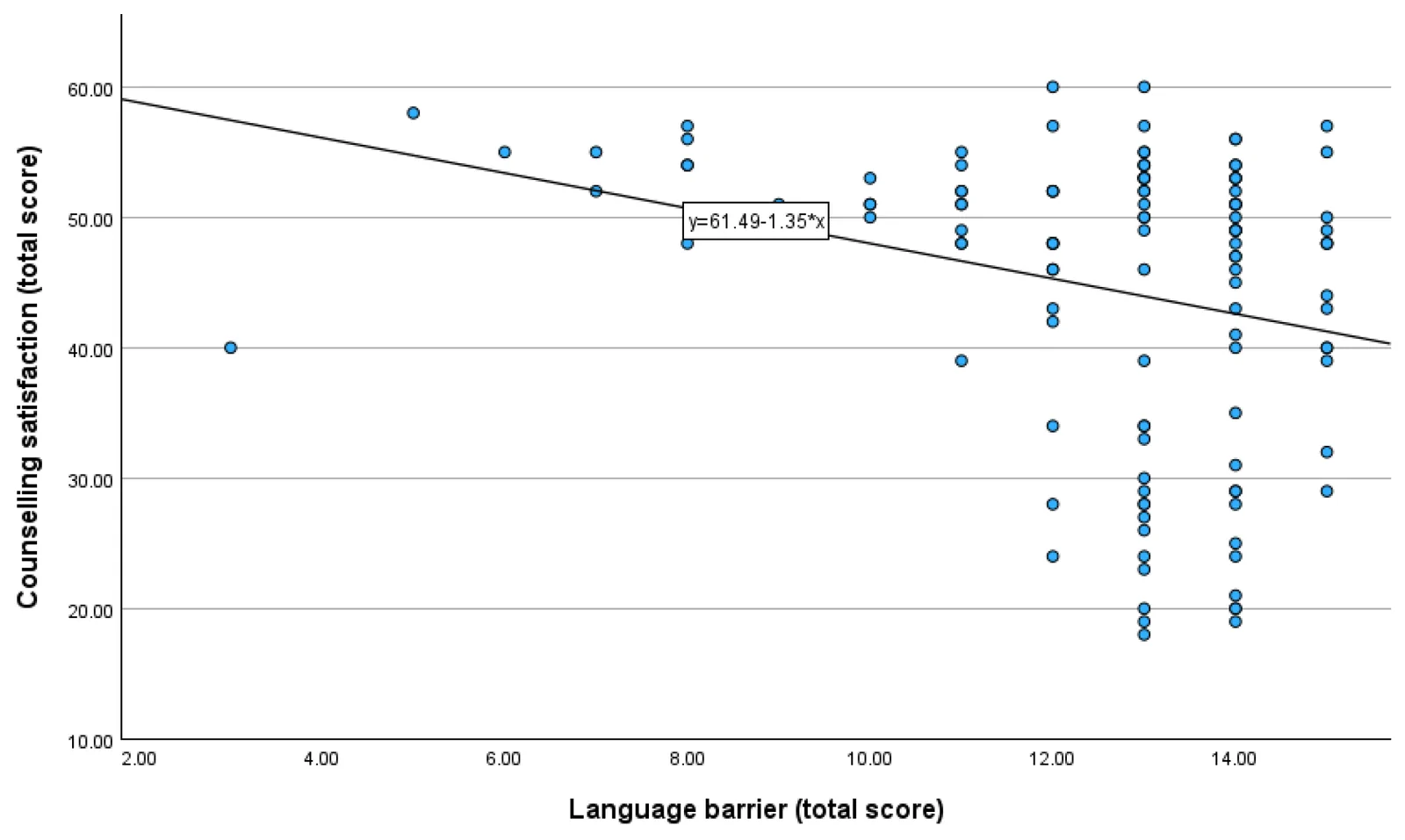
Scatterplot of counselling satisfaction and language barriers. The regression line indicates a significant negative correlation (R^2^ = 0.07, *p* < 0.01).

**Figure 4:**
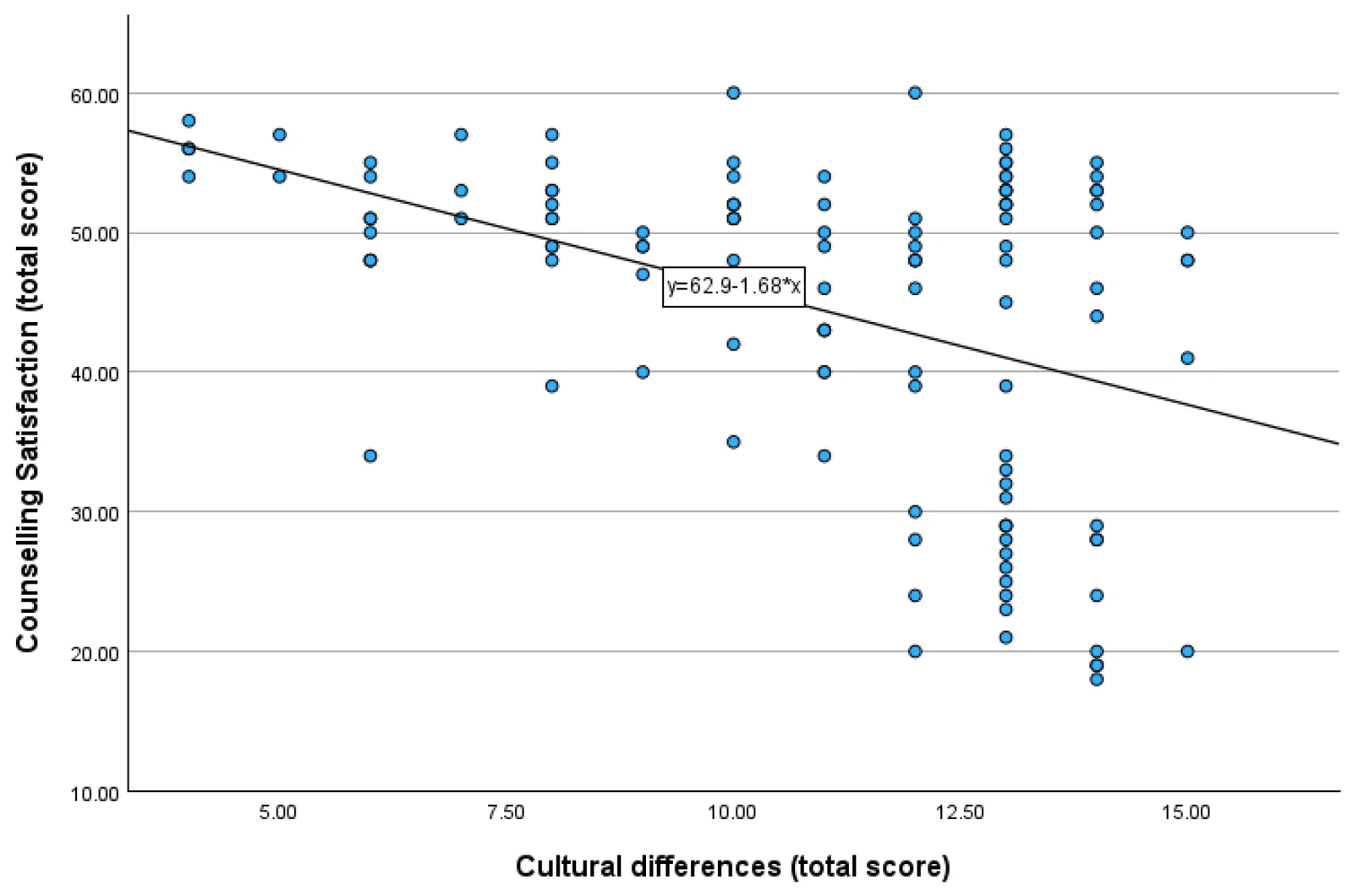
Scatterplot of counselling satisfaction and cultural differences. The regression line indicates a significant negative correlation (R^2^ = 0.19, *p* < 0.01).

**Figure 5:**
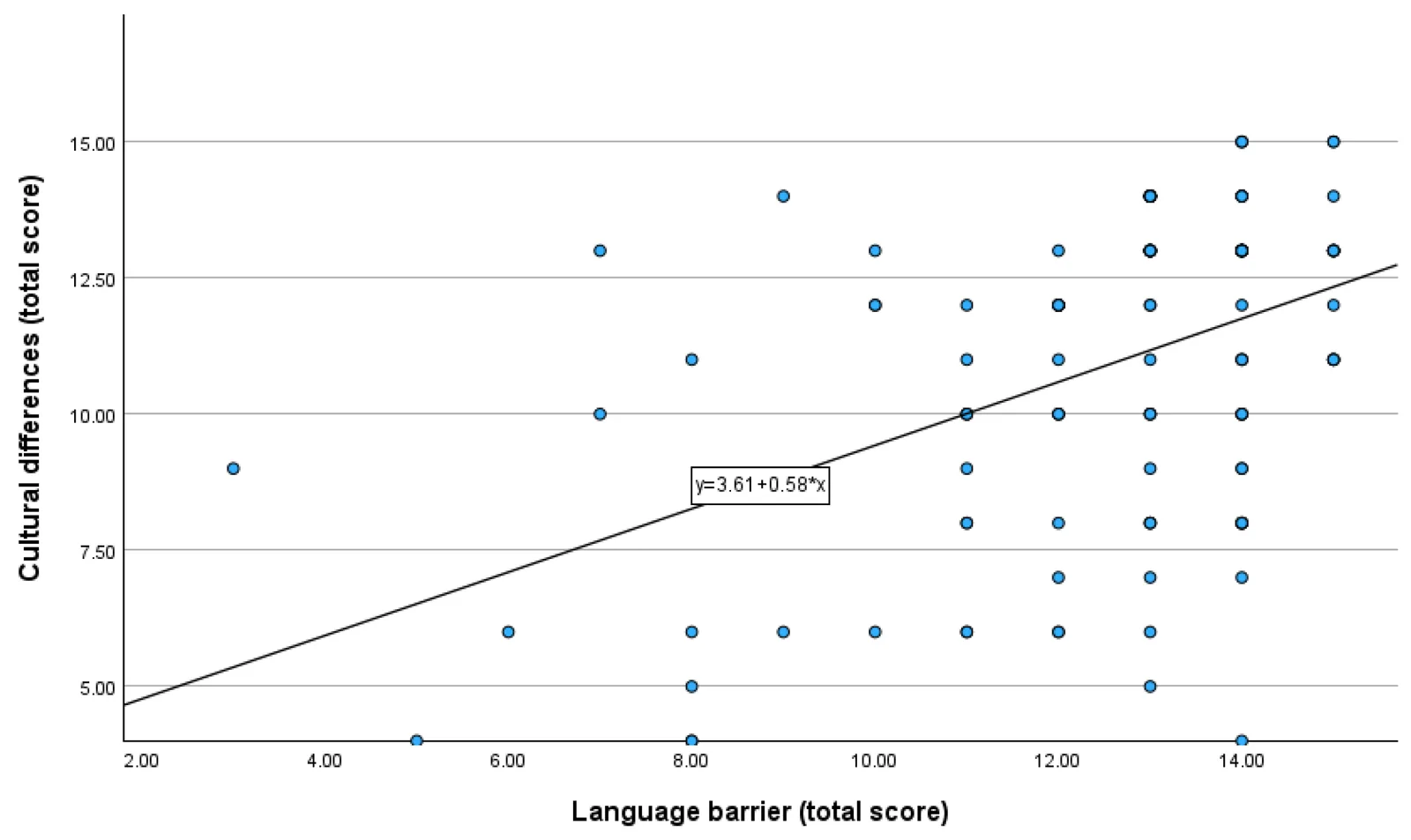
Scatterplot of language barriers and cultural differences. The regression line indicates a significant positive correlation (R^2^ = 0.20, *p* < 0.01).

Correlational analysis between satisfaction with counselling, language barriers, and differences between cultures was performed, and the correlation matrix is shown in Table 11. It was revealed that counselling satisfaction was negatively correlated with language barriers (r = −0.266, *p* < 0.01) and cultural differences (r = −0.435, *p* < 0.01), whereas language barriers had a positive effect on cultural differences (r = 0.444, *p* < 0.01). These relationships are further illustrated in scatterplots (Figure 3, Figure 4 and Figure 5).

#### 3.1.5. Regression Analysis of Counselling Satisfaction, Language Barriers, and Cultural Differences

To examine the unique associations of perceived language barriers and cultural differences with counselling satisfaction, a multiple linear regression model was fitted with counselling satisfaction as the dependent variable. The final model included perceived language barriers and cultural differences as predictors. No demographic covariates were included in this model; therefore, the regression coefficients should be interpreted as associations within the present sample rather than as fully adjusted effects. The adjusted R^2^ was 18.2%, indicating that the model accounted for 18.2% of the variance in counselling satisfaction. The overall regression model was statistically significant (F = 14.831, *p* < 0.001). Prior to the regression analysis, the assumptions of normality of residuals, homoscedasticity, multicollinearity, and independence of errors were examined. The VIF values were below 5, indicating no evidence of problematic multicollinearity. The details are presented in Table 12.

To examine the unique associations of perceived language barriers and cultural differences with counselling satisfaction, a multiple linear regression model was fitted with counselling satisfaction as the dependent variable. The final model included perceived language barriers and cultural differences as predictors. No demographic covariates were included in this model; therefore, the coefficients should be interpreted as associations within the present sample rather than as fully adjusted effects.

Based on the unstandardized regression coefficients, the regression equation can be expressed as follows: Counselling Satisfaction = 66.997 − 0.462 × Language Barrier − 1.526 × Cultural Difference. The negative coefficients indicate that greater perceived language barriers and cultural differences were associated with lower counselling satisfaction. However, only cultural differences showed a statistically significant unique association in the final model. Among the predictors examined, cultural differences therefore showed the strongest negative association with counselling satisfaction.

The regression model explained 18.2% of the variance in counselling satisfaction (R^2^ = 0.182), indicating that additional factors not examined in the present study are likely to contribute to counselling experiences. Therefore, the findings should be interpreted as partial explanations rather than comprehensive predictors of counselling satisfaction.

Contrary to expectations, perceived language barriers were no longer statistically significant after cultural differences were included in the regression model. This finding may indicate that perceived language difficulties overlap with broader cultural adaptation experiences. To provide greater contextual understanding of this result, qualitative interviews explored participants’ experiences of communicating in a second language during counselling sessions.

### 3.2. Qualitative Results

The results of the qualitative semi-structured interviews are presented in Appendix C. Building on the quantitative findings, which indicated that cultural differences showed the strongest negative association with counselling satisfaction, this qualitative analysis offers a more nuanced, context-rich exploration of the factors shaping participants’ experiences. While the sample size is smaller (n = 10), the interviews reveal how not only cultural differences but also language barriers influence users’ feelings and evaluations of counselling. In addition, the findings highlight other influential dimensions—such as counsellors’ attitudes, their patience, and their ability to build bonds with clients, as well as the alignment between clients’ goals and therapeutic approaches—that extend beyond the quantitative scope. Drawing on participants’ real-life experiences, the analysis also captures their suggestions for improving counselling services, including culturally sensitive practices, greater flexibility in service delivery, and enhanced accessibility.

The participants received counselling services in diverse settings: three in Canada, two in Australia, one in Germany, and four in the United Kingdom. Most sessions were conducted in English, with one participant in Australia receiving counselling in Chinese from a bilingual, dual-cultural counsellor—an experience that provided particularly rich insights into the potential benefits of cultural and linguistic alignment in therapy. The number of sessions reported ranged from one-time consultation to multiple meetings, offering a variety of perspectives shaped by different levels of exposure to counselling. Participants also reported different ways of learning about the availability of university counselling services. The most common channels included prior personal knowledge, referrals from doctors, recommendations by friends, and university publicity.

#### 3.2.1. Cultural Distance Reduced Counselling Comfort

Many participants reported that cultural differences created barriers to feeling understood by counsellors. Differences in family values, academic expectations, and help-seeking norms often resulted in perceived misunderstandings during counselling sessions. One participant explained: “My counsellor understood the theory, but not the pressure from my family. I felt I had to explain everything from the beginning.” These experiences help explain the strong negative association between perceived cultural differences and counselling satisfaction observed in the quantitative findings.

#### 3.2.2. Language as a Barrier

Several participants described language difficulties as obstacles to emotional expression. Communicating complex emotions in a second language required additional cognitive effort and occasionally led to misunderstandings. Some people said, “I knew what I wanted to say in Chinese, but I could not find the right words in English.” Participants often reported slowing down conversations or simplifying their emotional experiences when communicating in English.

#### 3.2.3. Language as a Facilitator

Interestingly, not all participants perceived language differences negatively. Several students reported that using English created emotional distance from painful experiences, making self-disclosure easier. One participant described this experience as follows: “Speaking English felt less emotional than speaking Chinese. It helped me discuss difficult experiences more calmly.” This finding helps explain why language barriers did not remain statistically significant in the regression model. For some students, second-language use functioned simultaneously as both a challenge and a source of psychological safety.

#### 3.2.4. Alliance-Related Experiences

Participants consistently highlighted the importance of alliance-related experiences, including empathy, trust, goal agreement, and collaborative problem solving. Participants described these experiences in the following terms: “The counsellor genuinely listened and did not judge me. That mattered more than language.” “Once I trusted my counsellor, language became less important.” These findings suggest that alliance-related experiences may help students navigate language and cultural challenges, although the present study did not quantitatively test mediation or moderation effects.

### 3.3. Integration of Quantitative and Qualitative Findings

To achieve methodological integration, quantitative and qualitative findings were compared using a joint display approach (Table 13). Rather than presenting the two datasets independently, the joint display was used to identify areas of convergence, complementarity, and explanation between statistical results and participants’ interview accounts. This approach enabled the qualitative findings to provide contextual explanations for quantitative associations, thereby generating a more comprehensive understanding of counselling satisfaction among Chinese international students. Qualitative findings both confirmed and extended the quantitative results. While quantitative analyses identified cultural differences as the strongest correlate of counselling satisfaction, qualitative interviews clarified how these differences were experienced in practice. Interviews further explained why language barriers did not remain significant in the regression model by revealing both negative and positive functions of second-language use. Together, these findings provide a more nuanced understanding of counselling experiences among Chinese international students.

**Figure 6:**
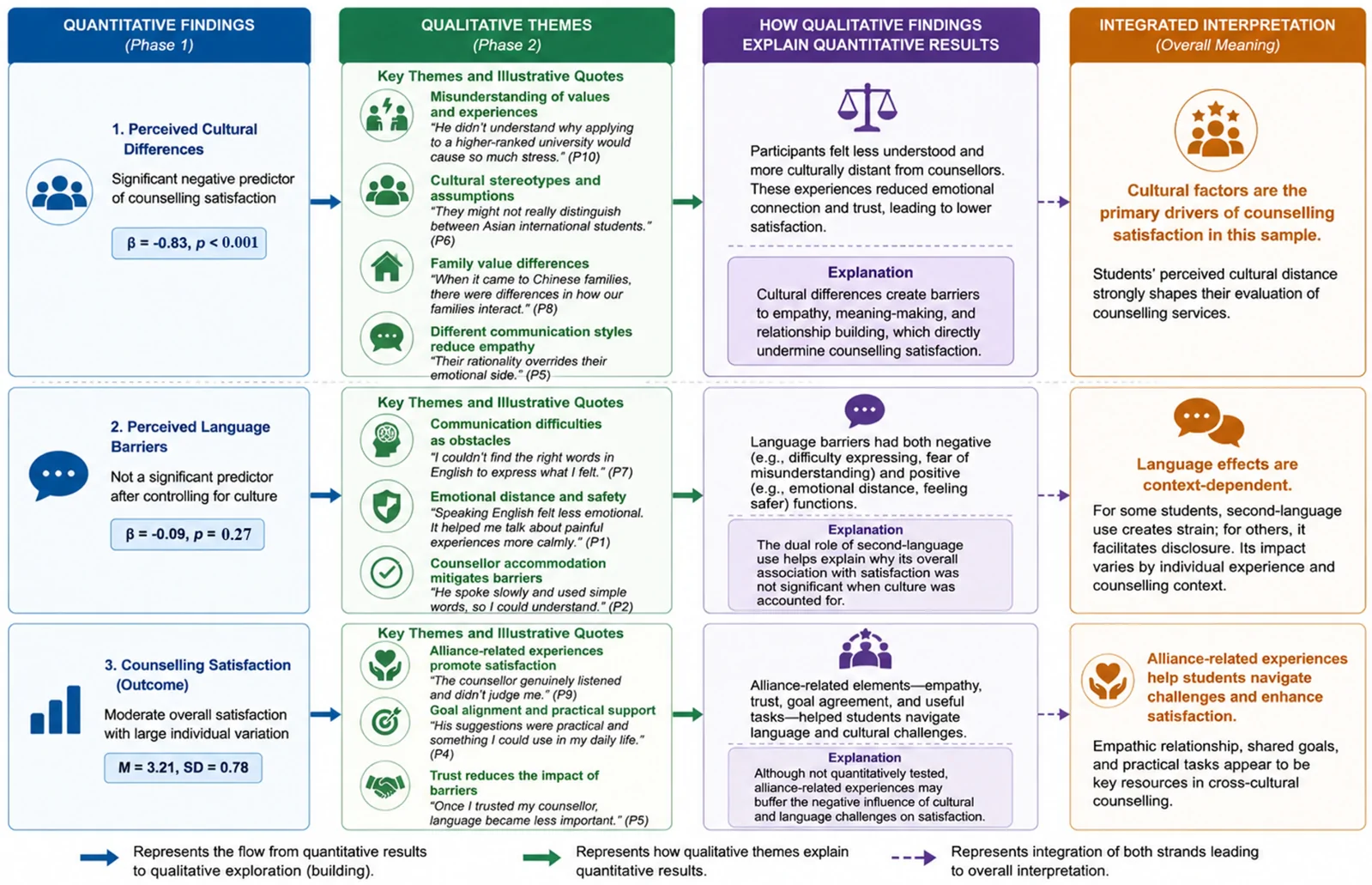
Joint display of quantitative and qualitative findings and integrated interpretation.

Overall, the integrated interpretation presented in Figure 6 demonstrates that quantitative and qualitative evidence complemented one another rather than operating independently. The quantitative analyses identified statistical patterns, whereas the qualitative findings explained how and why these patterns emerged in students’ lived experiences. Consequently, the explanatory sequential mixed-methods design provided a richer and more nuanced understanding of counselling satisfaction than either quantitative or qualitative methods alone.

## 4. Discussion

### 4.1. Principal Finding

The present study employed an explanatory sequential mixed-methods design to investigate counselling satisfaction among Chinese international students. Overall, three principal findings emerged. First, perceived cultural differences showed the strongest negative association with counselling satisfaction. Second, perceived language barriers were not independently associated with counselling satisfaction after cultural differences were controlled for. Third, qualitative findings suggested that alliance-related experiences—including empathy, trust, collaborative goal setting, and practical therapeutic support—may help explain why some students reported positive counselling experiences despite language and cultural challenges. Together, these findings provide a more nuanced understanding of counselling experiences among Chinese international students.

An important contribution of this study lies in the integration of quantitative and qualitative evidence. Whereas quantitative analyses identified statistical associations, qualitative interviews provided contextual explanations for these findings. In particular, interviews clarified why language barriers did not remain statistically significant after accounting for cultural differences and illustrated how culturally responsive communication practices may improve counselling experiences. These findings demonstrate the added value of the explanatory sequential mixed-methods design.

### 4.2. Language Mechanisms: Cognitive Load and Affective Distance in a Mixed Pattern of Effects

Quantitatively, perceived language barriers show a modest negative correlation with satisfaction. However, when cultural differences are entered into the regression, the language coefficient is not significant. There are several theoretically grounded reasons for this. First, the qualitative interviews demonstrate heterogeneity. Several participants described clear cognitive load effects. They struggled to retrieve precise words, could not easily convey fine-grained details, and felt that their emotions were not conveyed by their words. Second, others experienced little impact because sessions centred on being listened to, supported by non-verbal attunement and deliberate accommodation by counsellors. Additionally, the sample composition likely attenuated language effects through restriction of range. Nearly half of participants reported proficient or native-level fluency in the university’s language. Where proficiency is high and counsellors routinely adjust pacing and vocabulary, the cognitive load pathway is less likely to depress satisfaction, which again reduces the size of the language effect. Together, these reasons support the current result that the unique language effect is small.

Finally, the interviews showed mechanisms by which language effects are moderated by counsellor behaviour. Slowing down, simplifying phrasing, checking understanding, and noticing emotional cues all reduce cognitive load and make room for meaning repair. Where such adjustments were perceived, language seemed to matter less. Where such adjustments did not occur, participants reported reduced efficiency, fear of being misunderstood, and intensification of negative effects during their attempt to narrate trauma. Taken together, these patterns indicate that the effect of language on satisfaction is unstable across students and sessions, with substantial variability driven by whether cognitive load or affective distance is activated and by the extent of counsellor accommodation.

Contrary to our initial expectation, perceived language barriers did not remain statistically significant after cultural differences were included in the regression model. The qualitative findings provide several possible explanations for this result. First, language barriers may partially overlap with broader cultural adaptation processes. Second, participants reported that counsellors’ communication strategies, including slowing speech, checking understanding, and adapting explanations, substantially reduced communication difficulties. Third, some students perceived communicating in English as emotionally less intense than communicating in Chinese, allowing greater psychological distance when discussing sensitive experiences. These findings suggest that the influence of language is context-dependent and may differ across counselling settings and individual communication preferences.

### 4.3. Cultural Congruity and Cultural Humility as Primary Drivers of Satisfaction

The finding that perceived cultural differences were more strongly associated with counselling satisfaction than language barriers is consistent with previous research emphasizing the importance of culturally responsive counselling. Rather than reflecting differences in language alone, participants frequently described broader challenges involving family expectations, communication styles, emotional expression, and culturally shaped attitudes toward mental health. These findings suggest that cultural understanding may play a more influential role than linguistic proficiency in shaping counselling experiences among Chinese international students.

The cultural lens receives the strongest empirical support in this study. Perceived cultural differences showed the clearest and most consistent link with lower counselling satisfaction, and this association remained robust in the multivariate model. In practical terms, greater perceived cultural distance went hand in hand with noticeably lower satisfaction. The qualitative accounts supply concrete content for these concepts. Students described moments when counsellors did not understand the academic prestige economy that structured their stress, relied on broad regional stereotypes, or approached sessions with a communication style that felt argumentative rather than empathic. These are instances of low cultural congruity that undermine alliance formation and depress satisfaction. In contrast, when counsellors showed curiosity about family dynamics, slowed down to ask about context, or shared a similar cultural background, participants reported easier rapport, less need for lengthy explanations, and a feeling of being known.

It is noteworthy that perceived cultural differences did not vary reliably by age, duration abroad, or language proficiency in the quantitative analyses. This suggests an interactional experience in counselling rather than a stable trait. In other words, cultural differences are registered in the room through the way counsellors understand academic pressure, family roles, and acceptable emotional expression.

A further observation helps to explain why culture emerges as a primary predictor. Perceived language barriers were positively associated with perceived cultural differences. This pattern points to conceptual overlap, which is visible in the interview material. Many misunderstandings were not about vocabulary or grammar but about reasoning styles, assumptions about family obligations, and expectations of what a counsellor should do in a session. These are cultural frames within which words are interpreted. When that frame is misaligned, students may also experience the language as more effortful. In the regression model, once this shared variance is accounted for by the cultural difference measure, the unique contribution of language barriers becomes small, which is exactly what the non-significant language coefficient indicates.

Two additional interpretive points reinforce this reading. First, the qualitative material indicates that culture exerts a more immediate influence on satisfaction than language and therefore functions as the more proximal driver. When the cultural fit is good or when counsellors practice cultural humility, students can tolerate modest language strain and still evaluate sessions positively. When cultural congruence is poor, even fluent exchanges can feel off-target because meanings and role expectations diverge. Second, the same material shows how the working alliance operates within the cultural frame. Clear goals, an empathic bond, and practical tasks often restored momentum even when students perceived distance. Where these alliance elements were thin, the effects of cultural distance were amplified.

### 4.4. Alliance-Related Experiences as a Qualitative Explanatory Context

Although the present study did not quantitatively measure the working alliance, interview findings consistently highlighted alliance-related experiences—including empathy, trust, collaborative goal setting, and practical therapeutic support—as important aspects of positive counselling experiences. Therefore, rather than demonstrating mediation or buffering effects, the present findings suggest that alliance-related processes may help explain how some students successfully navigated language and cultural challenges during counselling. Future research should employ validated measures of the working alliance to examine these relationships more rigorously.

### 4.5. Exploratory Implications from Participants

Several practical implications emerge from the present findings. First, universities should strengthen culturally responsive counselling training by increasing counsellors’ understanding of Chinese cultural values, family expectations, and help-seeking beliefs. Second, counsellors should adopt communication strategies that reduce language-related difficulties, including checking understanding, using plain language, and encouraging clarification. Third, universities may consider providing multilingual counselling resources or interpreter support where appropriate. Finally, training programmes should emphasize alliance-related skills such as empathy, collaborative goal setting, and trust building, which participants consistently identified as important components of satisfying counselling experiences.

1. Advice for counsellors.

Participants emphasized a friendly and encouraging stance, stronger cross-cultural understanding, communication calibrated to the client’s language ability, adaptation to cultural and developmental background, and listening with individualized analysis. These preferences help to account for the variation in satisfaction observed across interviews. Warmth and careful listening support the bond element of the working alliance and make it easier to persist through moments of imprecision. Curiosity about family roles, academic pressures, and social values reduces the sense of cultural distance that emerged as the strongest quantitative predictor of lower satisfaction. Adjustments in pace and vocabulary and checks of understanding lower processing demands, which clarifies why some participants experienced little impact of language while others described a clear strain. Tailoring approaches to background aligns goals and makes concrete tasks feel doable beyond the session, consistent with reports of continued use of techniques.

2. Advice for services.

Participants wanted to know about and choose counsellors, to have shorter waits and easier access, to receive clearer communication about available support, to have more flexible session lengths and formats, and to see more diverse counselling resources, including translation assistance when native language provision is not feasible. These requests speak directly to the mechanisms identified above. Choice increases the likelihood of cultural congruity from the outset and reduces explanatory burden in early meetings. Shorter waiting times and clearer routes into care encourage earlier help-seeking, which allows communication adjustments and alliance building before distress escalates. Flexibility in session structure makes it more feasible to teach and rehearse practical strategies that transfer to daily life. Diversifying resources and offering translation support reduce misunderstandings when language demands are high and lessen perceived cultural distance. Together, the counsellor level and service level changes address the routes through which culture and language affected satisfaction in this sample.

### 4.6. Strengths and Limitations

This study has several strengths. It is among the few studies to investigate counselling satisfaction among Chinese international students using an explanatory sequential mixed-methods design. The integration of quantitative and qualitative findings provided richer insights than either method alone and highlighted the complexity of counselling experiences in cross-cultural contexts.

Several limitations should be acknowledged. First, the use of non-probability snowball sampling may have introduced self-selection and network-related sampling bias and may limit the representativeness of the quantitative sample. Participants who were more connected to relevant social networks or more willing to discuss counselling experiences may have been more likely to participate. Second, the qualitative interview participants were self-selected from survey respondents who volunteered for follow-up interviews. Although purposive selection was subsequently used to maximize variation in gender, host country, counselling satisfaction, and language proficiency, self-selection may still have favoured participants with stronger or more salient counselling experiences.

Third, the quantitative sample was not sufficiently balanced across demographic categories. Female participants accounted for approximately 80% of the sample, while some age and language-proficiency groups contained very few participants. These imbalances reduce statistical power for subgroup comparisons and limit the generalizability of the findings. In addition, the regression model included language barriers and cultural differences as the primary predictors and did not include demographic control variables such as gender or duration of stay. The resulting estimates should therefore be interpreted as associations within the present sample rather than comprehensive explanations of counselling satisfaction.

Fourth, the language barrier and cultural difference measures were developed specifically for this study. Although both subscales demonstrated high internal consistency in the present sample, internal consistency does not establish construct validity. Exploratory and confirmatory factor analyses were not conducted because these measures were exploratory, newly developed short scales, and the available sample was limited. Future studies should validate these measures in larger and independent samples using factor-analytic and other validity procedures.

Fifth, the qualitative interviews lasted approximately 15–25 min. Although this duration allowed participants to describe experiences directly relevant to the quantitative findings, it may have restricted the depth and breadth of their accounts. The qualitative sample was also relatively small and was selected for explanatory purposes rather than statistical representation. The findings should therefore be interpreted as contextual explanations rather than as exhaustive accounts of Chinese international students’ counselling experiences.

Sixth, participants were studying in different host countries, including the United Kingdom, Canada, Australia, and Germany. Differences in university counselling systems, service accessibility, counsellor training, and language provision across these settings may have influenced participants’ experiences. These contextual differences were not examined as independent predictors in the present analysis and therefore represent an additional source of unexplained variation.

Seventh, the study relied primarily on self-reported perceptions of counselling satisfaction, language barriers, and cultural differences. Such measures may be affected by recall, response style, or participants’ subjective interpretation of their counselling experiences. The cross-sectional design also prevents conclusions about temporal or causal relationships among the variables.

Finally, although alliance-related experiences emerged consistently in the qualitative data, the working alliance was not measured using a validated quantitative instrument. Accordingly, the present study cannot establish whether the working alliance statistically mediates or moderates the associations between language barriers, cultural differences, and counselling satisfaction. The alliance-related findings should therefore be interpreted as qualitative explanatory evidence and as a basis for future hypothesis testing rather than as evidence of empirically established mediation or moderation.

Future research should recruit larger and more diverse samples using probability-based sampling where feasible. Longitudinal and cross-cultural comparative studies would further clarify how counselling experiences evolve over time and across host countries. Future studies should also incorporate validated measures of the working alliance and culturally responsive counselling competencies to better understand the mechanisms underlying counselling satisfaction among international students.

In conclusion, the present study demonstrates that counselling satisfaction among Chinese international students is shaped primarily by perceived cultural differences rather than language barriers alone. Qualitative findings further indicate that alliance-related experiences, including empathy, trust, collaborative goal setting, and practical therapeutic support, may help students navigate cross-cultural counselling more effectively. These findings highlight the importance of integrating culturally responsive counselling practices with alliance-building strategies to improve mental health support for international students.

## 5. Conclusions

This study employed an explanatory sequential mixed-methods design to investigate counselling satisfaction among Chinese international students, with particular attention to perceived language barriers, cultural differences, and alliance-related experiences. The quantitative findings indicated that perceived cultural differences showed the strongest negative association with counselling satisfaction, whereas perceived language barriers were not independently associated with satisfaction after cultural differences were taken into account. Qualitative findings further demonstrated that students’ counselling experiences were shaped by complex interactions among cultural understanding, communication practices, and counsellor responsiveness. Together, these findings provide a more comprehensive understanding of counselling satisfaction within cross-cultural university counselling contexts.

Although the present study did not quantitatively measure the working alliance, qualitative findings consistently highlighted alliance-related experiences—including empathy, trust, collaborative goal setting, and practical therapeutic support—as important elements associated with positive counselling experiences. Accordingly, the findings should not be interpreted as evidence of mediation or buffering effects. Rather, they suggest that alliance-related experiences may help explain how some Chinese international students navigate language and cultural challenges during counselling. The study also contributes to the growing literature on international students’ mental health by demonstrating the value of integrating quantitative and qualitative evidence. Rather than relying solely on statistical associations, the explanatory sequential mixed-methods design enabled interview findings to contextualize and extend quantitative results, thereby providing a more nuanced understanding of counselling experiences among Chinese international students.

From a practical perspective, the findings suggest that universities should strengthen culturally responsive counselling practices by improving counsellors’ understanding of Chinese cultural values, enhancing culturally sensitive communication skills, and promoting alliance-related counselling practices such as empathy, collaborative goal setting, and trust building. These approaches may contribute to improving counselling experiences for international students from diverse cultural backgrounds. Although exploratory and cross sectional, the study offers a coherent account of when and why language and culture matter for satisfaction in university settings and how their influence travels through alliance processes. Future research should recruit larger and more diverse samples, include validated measures of the working alliance, and further examine counselling experiences across different host countries and counselling contexts. Longitudinal and cross-cultural comparative studies would also help clarify how counselling satisfaction develops over time and whether alliance-related experiences operate similarly across different international student populations.

By integrating quantitative analyses with qualitative explanations, this study extends current understanding of counselling satisfaction beyond simple statistical relationships. The mixed-methods approach enabled the identification of contextual factors that would not have been apparent through quantitative analysis alone, illustrating the importance of combining numerical evidence with participants’ lived experiences when investigating culturally complex counselling processes. Overall, the present findings underscore the importance of culturally responsive counselling and highlight the value of explanatory sequential mixed-methods research for advancing understanding of international students’ counselling experiences in increasingly diverse higher education settings.

## Figures and Tables

**Table 1:** Demographic characteristics of participants with valid questionnaires (N = 125).

Category	Group	N	Valid Percentage (%)	Cumulative Percentage (%)
Age	18–25 years	77	61.6	61.6
	26–30 years	45	36	97.6
	>31 years	3	2.4	100
Gender	Female	100	80	80
	Male	25	20	100
Nationality and Native Language	Chinese, Mandarin	125	100	100
Duration of Stay	<6 months	14	11.2	11.2
	6 months–12 months	58	46.4	57.6
	>1 year	53	42.4	100
Language Proficiency	Very Limited	1	0.8	0.8
	Limited	19	15.2	16
	Fair	45	36	52
	Proficient	59	47.2	99.2
	Native Level	1	0.8	100

**Table 2:** Counselling satisfaction scores of Chinese international students (N = 125).

	N	Min	Max	M	SD
Satisfaction	125	18	60	44.528	11.18436

**Table 3:** Results of *t*-Test of differences in counselling satisfaction among Chinese international students (N = 125).

	Group	Case	Mean	SD	t
Gender	Female	100	43.98	11.33242	−1.096
	Male	25	46.72	10.50206	

**Table 4:** ANOVA of differences in counselling satisfaction among Chinese international students (N = 125).

Category	Group	N	M	SD	F	LSD
Age	18–25 years	77	46.8312	9.35893	5.317 **	3 < 2 < 1
	26–30 years	45	41.3333	13.08712		
	>31 years	3	33.3333	6.1101		
Duration of Stay	<6 months	14	40.3571	14.55814	2.31	\
	6 months–12 months	58	46.6034	9.42177		
	>1 year	53	43.3585	11.70978		
Language Proficiency	Very Limited	1	55	\	0.395	\
	Limited	19	43.4737	11.25645		
	Fair	45	43.6889	11.31938		
	Proficient	59	45.339	11.2887		
	Native Level	1	44	\		

Note. ** *p* < 0.01

**Table 5:** Language barrier scores of Chinese international students (N = 125).

	N	Min	Max	M	SD
Language Barrier	125	3	15	12.568	2.2046

**Table 6:** Results of *t*-test of differences in language barriers among Chinese international students (N = 125).

Category	Group	N	Mean	SD	t
Gender	Female	100	12.7	2.16725	1.343
	Male	25	12.04	2.31805	

**Table 7:** ANOVA of differences in language barriers among Chinese international students (N = 125).

Category	Group	N	M	SD	F	LSD
Age	18–25 years	77	12.5714	1.94955	0.398	\
	26–30 years	45	12.4889	2.63389		
	>31 years	3	13.6667	1.52753		
Duration of Stay	<6 months	14	12.6429	2.40535	0.281	\
	6 months–12 months	58	12.7069	1.91951		
	1 year	53	12.3962	2.45998		
Language Proficiency	Very Limited	1	6	\	4.235 **	\
	Limited	19	13.5263	1.21876		
	Fair	45	12.7111	1.90242		
	Proficient	59	12.2203	2.42876		
	Native Level	1	15	\		

Note. ** *p* < 0.01.

**Table 8:** Cultural difference scores of Chinese international students (N = 125).

	N	Min	Max	M	SD
Cultural Difference	125	4	15	10.92	2.88936

**Table 9:** Results of *t*-test of differences in cultural differences among Chinese international students (N = 125).

Category	Group	N	Mean	SD	t
Gender	Female	100	10.83	2.99176	−0.695
	Male	25	11.28	2.45832	

**Table 10:** ANOVA of differences in cultural differences among Chinese international students (N = 125).

Category	Group	N	M	SD	F	LSD
Age	18–25 years	77	10.5065	2.77025	2.215	\
	26–30 years	45	11.5556	3.07154		
	>31 years	3	12	1		
Duration of Stay	<6 months	14	11.2143	3.35533	1.467	\
	6 months–12 months	58	10.4483	2.76045		
	>1 year	53	11.3585	2.87626		
Language Proficiency	Very Limited	1	6	\	1.758	\
	Limited	19	11.9474	2.36816		
	Fair	45	10.6667	2.94649		
	Proficient	59	10.8136	2.92123		
	Native Level	1	14	\		

**Table 11:** Correlation analysis of counselling satisfaction, language barriers, and cultural differences.

	Satisfaction	Language Barrier	Cultural Difference
Satisfaction	1		
Language Barrier	−0.266 **	1	
Cultural Difference	−0.435 **	0.444 **	1

Note. ** *p* < 0.01.

**Table 12:** Regression analysis results of counselling satisfaction, language barriers, and cultural differences.

	B	t	Significance	95.0% CI for B		VIF
				Lower CI	Upper CI	
(Intercept)	66.997	12.393 ***	<0.001	56.295	77.698	
Language Barrier	−0.462	−1.005	0.317	−1.372	0.448	1.246
Cultural Difference	−1.526	−4.350 ***	<0.001	−2.22	−0.832	1.246

Note. *** *p* < 0.001.

**Table 13:** Integration of quantitative and qualitative results.

Quantitative Finding	Qualitative Explanation	Integrated Interpretation
Cultural differences significant	Students described misunderstandings	Culture strongly influences satisfaction
Language not significant	Some students viewed English positively	Language effects are context-dependent
Satisfaction varied	Trust and empathy frequently mentioned	Alliance-related experiences may explain differences

## Data Availability

The data presented in this study are available on request from the corresponding author. The data are not publicly available due to privacy and ethical considerations, as the dataset contains potentially identifiable information related to participants’ counselling experiences and personal experiences.

## Appendix A

### Appendix A.1. Section A: Demographics (6 Items Single Choice Unless Noted)

1. What is your age? ☐ 18–25 years ☐ 26–30 years ☐ above 31 years

2. Self-identified gender: ☐ Female ☐ Male ☐ Non-binary/Other ☐ Prefer not to say

3. What is Country/region of origin and native language (first language)?

☐ China and Chinese (Mandarin)

☐ Other (If you choose this, you are not eligible for this questionnaire. Thank you for your participation.)

4. Duration of study in the host country/region abroad:

☐ <6 months ☐ 6–12 months ☐ >1 year

5. Self-rated proficiency in the university’s main language (e.g., English):

☐ Very Limited ☐ Limited ☐ Fair ☐ Proficient ☐ Native Level

### Appendix A.2. Section B: Attitudes Toward Seeking Professional Psychological Help ATSPPH-SF

(5-point Likert: 1 Strongly disagree, 2 Disagree, 3 Neither agree nor disagree 4 Agree 5 Strongly agree)

6. If I felt stressed or depressed, I would be willing to talk to a professional counsellor.

7. I believe counselling can help students solve psychological problems.

8. I would feel comfortable if my friends knew I was seeing a counsellor.

9. People from my culture generally approve of seeking professional mental-health help.

### Appendix A.3. Section C: Satisfaction with Therapy & Therapist Scale-Revised (STTS-R)

(5-point Likert: 1 Strongly disagree, 2 Disagree, 3 Neither agree nor disagree 4 Agree 5 Strongly agree, except Q10.)

10. How many counselling sessions have you attended?

☐ 1 ☐ 2–3 ☐ 4 or more

11. I am satisfied with the quality of the therapy I received.

12. This therapy met my needs.

13. The therapist listened to what I was trying to say.

14. The therapist provided an adequate explanation regarding my therapy.

15. The therapist was not negative or critical toward me.

16. The therapist was friendly and warm toward me.

17. I felt free to express myself.

18. I was able to focus on what was of real concern to me.

19.The therapist seemed to understand what I was thinking and feeling.

20. I am now able to deal more effectively with my problems.

21. I would return to the clinic if I need any help.

22. I would recommend the service to other international students.

### Appendix A.4. Section D: Language & Cultural Barriers (5-Point Likert)

(5-point Likert: 1 Strongly disagree, 2 Disagree, 3 Neither agree nor disagree 4 Agree 5 Strongly agree)

23.Speaking in a second language made it hard to express my feelings in counselling.

24. I was worried the counsellor might misunderstand me because of language.

25. I sometimes needed to translate mental-health terms mentally before answering.

26. Cultural differences (values, communication style) made counselling less comfortable.

27. I felt the counsellor sometimes compared my behaviour to that of local (majority-culture) students.

28. The counsellor did not show understanding of the historical and social context affecting people from my culture.

## Appendix B

Q1. Please briefly introduce your basic information. (For example: nationality, native language, the country and academic stage you are currently or previously studied in.)

Q2. Have you used the psychological counseling service provided by your school?

Q3. How did you learn about the psychological counseling service offered by the school? (Such as the school’s official website, recommendations from classmates, introductions from teachers or school doctors, social media, etc.)

Q4. Before your first counseling session, what expectations or imagination did you have for this service? Q5. How are the actual consulting experiences similar or different from the initial expectations?

Q6. Overall, how do you evaluate your experience of psychological counseling?

Q7. Before and after receiving psychological counseling, did your views or attitudes towards counseling change?

Q8. During the counseling process, which language did you mainly use?

Q9. Do you think the use of language has had an impact on the counseling process or the counseling outcome?

Q10. Has language ever been a barrier when expressing emotions, thoughts, or specific experiences?

Q11. Do you feel that there are cultural differences between you and the counselor?

Q12. If there are cultural differences, do they affect your understanding, trust, or comfort in communication during the counseling session?

Q13. From your perspective, in which aspects is the current psychological counseling service provided by the school doing well? Q14. What aspects do you think still need improvement for the campus psychological counseling services, especially in terms of supporting international students?

Q15. If the psychological counselor is going to provide services to students from different cultural and linguistic backgrounds, what suggestions would you give them?

Q16. Are there any other experiences, feelings, or thoughts related to the campus psychological counseling services that were not covered in the previous questions but that you would like to add?

## Appendix C

### Appendix C.1. Theme 1: Real Counselling Experience (Working Alliance)

Participants described their actual counselling experiences, reflecting on how they felt during the sessions, how they evaluated them, and whether their expectations were met. In discussing these experiences, they often highlighted the specific tasks and strategies introduced in counselling, the bond developed with their counsellors, and the degree to which their goals aligned with those of the counsellors. These aspects together shaped how participants evaluated the quality and effectiveness of the university counselling services they had received so far.

#### Appendix C.1.1. Goals

Expectations varied widely among participants. Some sought emotional relief and understanding, while others hoped for concrete solutions. Within this subtheme, the coding captured three aspects: participants’ specific goals, whether they felt their goals were achieved, and cases where their goals were not achieved. These codes helped illustrate the diversity of experiences. A few participants reported that their goals were met early on, which led to a sense of satisfaction and motivation to continue. Others felt that their goals were only partially achieved, leaving them uncertain about the value of counselling. Still others reported that their goals were not achieved at all, which often resulted in disappointment and reduced satisfaction.

“I hoped he could listen to my problems and give professional advice.” (Participant 04)

“I just wanted a place to vent without moral judgment or worrying about future relationships.” (Participant 08)

“I wanted him to tell me the solution, like a doctor prescribing medicine.” (Participant 03)

Misalignment between expectations and actual counselling experience sometimes led to dissatisfaction. In contrast, when goals were aligned, participants reported more positive and productive sessions.

#### Appendix C.1.2. Bonds

The bonds between counsellors and clients are described as either positive or negative. The coding distinguished between these two types of experiences. Some participants felt that their counsellors were supportive, empathetic, and non-judgmental, which gave them a sense of safety and understanding.

“Having a professional who truly understands what you are describing makes you feel empathized with and supported.” (Participant 02)

“The counsellor patiently listened and even handed me tissues when I cried.” (Participant 07)

By contrast, others held negative feelings towards their counsellors. They reported a lack of trust or felt that the counsellor did not truly listen and instead rushed to provide solutions. Such negative bonds often discouraged participants from becoming fully involved in the counselling process.

“I felt that the counselor completely failed to hear my concerns or recognize the points that made me really upset. She just seemed focused on quickly giving me a solution and ending the session. As a result, I also wanted to just go along with it and finish as soon as possible. I was not truly engaged in the process myself.” (Participant 05)

#### Appendix C.1.3. Tasks

This subtheme has one code, that is, specific coping methods and strategies. Participants described how their counsellors introduced concrete techniques to help them manage emotions, as well as explained related psychological knowledge to support their use. Many valued the practical strategies that could be directly applied in daily life. They also noted that these strategies not only were effective during the counselling sessions but continued to help them afterwards, even in their current daily lives.

“I had no idea what a panic attack was. My counsellor explained it simply and gave me small methods to cope.” (Participant 02)

“He taught me some methods, such as effective deep breathing. For example, he asked me to look at the sides of a house and take one deep breath for each side. After he guided me through several rounds of deep breathing, I noticed that I gradually calmed down.” (Participant 07)

“I think he gave me one or two suggestions that I still use now.” (Participant 10)

Such guidance was valued for its immediate applicability and for helping participants feel more in control during distressing moments.

### Appendix C.2. Impact of Language Barriers on Counselling Experience

Participants reported varied experiences of how language influenced their counselling. For some, language barriers had little or no impact. For others, using a second language sometimes created a positive effect, while for some it became a major obstacle. In this theme, the coding captured these three types of impact—no impact, positive impact, and negative impact. Participants also reflected in detail on why language barriers shaped their experiences in these ways, offering insights into both the challenges and the unexpected benefits of language use in counselling.

#### Appendix C.2.1. No Significant Impact on Overall Effectiveness

This subtheme was supported by four codes: counselling content focused mainly on listening, non-verbal communication enhanced understanding, the content of counselling placed low demands on language ability, and counsellors adjusted their expression to match the client’s language level. Together, these factors explain why some participants felt that language barriers did not affect their overall satisfaction with counselling. They described counselling as being centred more on listening than speaking, supported by non-verbal cues such as tone and gestures. In addition, they thought the nature of the sessions did not require advanced language skills, and counsellors often slowed down, simplified their language, or checked for understanding. As a result, these participants perceived that language differences had little influence on the effectiveness of their counselling experience.

“In all the counselling sessions I have had—and I have had quite a few, as I just mentioned—I would say that over 95% of the time it was me talking while the counsellor was mainly listening. So even after attending so many sessions, I don’t feel that language has had much impact. For me, it was not a problem at all.” (Participant 02)

“So even if he didn’t fully understand why I was so upset, when he saw me crying so hard, he could at least tell that I was really distressed, and he made an effort to give me emotional feedback.” (Participant 07)

“This further reduced the demand for language in the counselling communication process.” (Participant 02)

“Because I was an international student, he could sense my language level from the way I spoke. So, when talking with me, he spoke very slowly and used daily conversation words, which made it easy for me to understand.” (Participant 02)

#### Appendix C.2.2. Creating Barriers

This subtheme was supported by eleven codes: limited vocabulary leading to imprecise expression, restricted expression of details, restricted emotional expression, differences in language logic leading to meaning deviations, incomplete reception of counsellors’ messages, counsellors’ misunderstanding of clients’ expressions, counsellors’ own language limitations affecting interaction, reduced efficiency of expression, worry about being misunderstood or communicating inefficiently due to language, lack of confidence in second-language expression, discomfort caused by heightened awareness of language identity, and language barriers intensifying negative emotions. Participants explained in detail why they felt language could become an obstacle in counselling. They described struggling to express emotions precisely, to capture important details, or to convey cultural differences. At the same time, they sometimes found it difficult to fully understand what the counsellor meant, and in some cases felt misunderstood. These difficulties not only slowed down communication but also affected how safe and comfortable participants felt in the sessions. For some participants, language barriers reduced their confidence and even worsened negative emotions, further complicating the counselling process.

“I had to tell the counsellor that I kept trembling, that I couldn’t sleep, and that I felt really bad inside. But when describing my symptoms like that, I worried that the counsellor might not understand me, or that I wasn’t explaining them accurately.” (Participant 07)

“But when I was using my second language, which I am not very good at, it greatly slowed down my speaking speed.” (Participant 03)

“It wasn’t just the feeling of not being able to explain myself clearly. It was more like my emotions were stuck somewhere and just couldn’t be conveyed.” (Participant 05)

“Sometimes the way I expressed myself didn’t really fit their language logic, so they couldn’t understand what I was saying.” (Participant 10)

“So when I recalled those traumas, it was already very painful. And having to think, in the middle of that pain, about how to describe the trauma precisely in my second language made it even harder. It felt as if my mouth were blocked, or as if someone were choking my neck.” (Participant 03)

#### Appendix C.2.3. Facilitating Counselling Effectiveness

This subtheme has one code: emotional protection from using a second language. Interestingly, a few participants preferred using English rather than their native language when discussing sensitive topics, as it created emotional distance and a sense of safety.

“Using English gives me a sense of security when talking about past wounds.” (Participant 04)

“When I talk about it in Chinese, I feel a bit ashamed. But when I talk about it in English, it feels much easier, almost as if there’s a layer of distance, and I can describe my inner thoughts quite normally.” (Participant 09)

### Appendix C.3. Impact of Cultural Background Differences on Counselling Experience

Participants expressed varied views on how cultural background differences shaped their counselling experiences. Some felt that cultural differences had little impact, while others reported that such differences created barriers to understanding. In the meantime, two participants whose counsellors shared similar cultural backgrounds described how this familiarity enhanced mutual understanding and made the counselling feel more supportive and effective.

#### Appendix C.3.1. No Noticeable Impact

Some participants felt that cultural differences did not significantly influence their counselling experiences. In some cases, they acknowledged that their counsellors came from different cultural backgrounds but did not see this as shaping the process in any meaningful way. In other cases, participants described encountering unexpected aspects of counselling but did not attribute these differences to culture, suggesting that they did not consciously link their experiences to cultural background. As a result, for these participants, cultural factors were not perceived as playing a major role in their satisfaction or evaluation of counselling.

“I feel that some psychological problems are not limited by national borders—everyone can experience them, and counsellors can also understand them.” (Participant 04)

“I think this is hard to judge, because you don’t know whether the difference comes from personality or from culture.” (Participant 09)

#### Appendix C.3.2. Creating Barriers

For some participants, cultural differences created obstacles in counselling. This subtheme was supported by four codes: differences in academic values affecting understanding, cultural stereotypes shaping counsellors’ judgments, family value differences influencing counsellors’ interpretations, and cross-cultural communication styles reducing empathy. Participants described feeling that their counsellors did not fully understand the academic pressures or family expectations they faced, which limited the counsellors’ ability to empathize. In some cases, they sensed that counsellors relied on cultural stereotypes, which led to misjudgment about their problems. Differences in communication style also made participants feel less emotionally connected, contributing to a sense of disconnect in the counselling relationship.

“He didn’t understand why applying to a higher-ranked university would cause stress. He just looked confused.” (Participant 10)

“They might not really distinguish between Asian international students, like whether someone is Chinese, Japanese, or Korean.” (Participant 06)

“When it came to talking about Chinese families, the patterns were quite different from theirs, so there were some differences in understanding the way Chinese families interact.” (Participant 08)

“In Germany, their rationality tends to override their emotional side. Even counsellors tend to rely on logic and argumentation, which I feel is not well suited to counselling” (Participant 05)

#### Appendix C.3.3. Facilitating Understanding

This subtheme was supported by two codes: similar cultural background enhancing understanding and empathy and similar cultural background reducing communication effort. Two participants whose counsellors shared similar cultural backgrounds reported that this familiarity made communication easier and more natural. They felt that they did not need to provide lengthy explanations about cultural values, family dynamics, or social pressures, because their counsellors already had an implicit understanding. This sense of shared background allowed participants to feel more fully understood and supported, which in turn enhanced their overall counselling experience.

“My counsellor is a Chinese living abroad. Since he had also received Western education, I felt he combined the strengths of both Chinese and Western approaches.” (Participant 01)

“I needed to spend much more time describing my childhood, education, and family experiences to my mentor. But when I talked with my counsellor, I only needed a few sentences, and he could understand without me explaining so much.” (Participant 03)

### Appendix C.4. Suggestions for Improvement from the Users’ Perspective

Participants shared a range of suggestions for improving counselling services. These suggestions covered both the personal qualities of counsellors and the structural organization of the service. Overall, participants emphasized that such improvements could make counselling more supportive and accessible for international students.

#### Appendix C.4.1. Areas for Counsellors to Improve

This subtheme was supported by five codes: having a friendly and encouraging attitude, enhancing cross-cultural understanding, adjusting communication styles to clients’ language ability, adjusting counselling approaches to clients’ cultural and developmental background, and prioritizing listening with individualized analysis. Participants believed that a welcoming and encouraging attitude could help clients relax and open up, especially those from different cultural backgrounds. At the same time, participants expressed that counsellors would benefit from improving their sensitivity to cultural differences, as well as adapting both their communication and therapeutic strategies to the client’s linguistic level and cultural context. Together, these suggestions include concrete ways in which counselling services could be made more accessible and effective for international students.

“It’s important to encourage the client, because most people who come for counselling are psychologically vulnerable or hurt. They hope to be encouraged rather than given advice.” (Participant 02)

“For Asian students like us, it would help if counsellors understood more about our country’s situation, the background we grew up with, or the kinds of ideas our parents passed on to us.” (Participant 05)

“First of all, counsellors should not rely on prior experience or stereotypes when conducting counselling. They should remind themselves that this case may be different from ones they have encountered before, and instead take a fully listening approach—step by step exploring what the client truly needs. It should not be about hearing a certain keyword and then applying a ready-made formula. The focus should be more on the client as an individual.” (Participant 07)

#### Appendix C.4.2. Feedback on Counselling Services

This subtheme was supported by five codes: granting clients the right to know about and choose their counsellors, reducing waiting times and improving accessibility, increasing publicity about available services, offering more flexible session lengths and formats, and diversifying counselling resources. Participants raised several concerns about the organization of counselling services. A common frustration was the lack of choice in selecting counsellors, which sometimes limited the sense of fitness and comfort in the counselling relationship. Long waiting times were also frequently mentioned, with participants emphasizing the need for more timely and accessible support. Another area for improvement was publicity: many felt that information about services was not widely or clearly communicated, which reduced awareness and uptake. Participants also expressed a wish for greater flexibility in session arrangements, including the option of longer or differently structured sessions. Finally, they called for a broader range of counselling resources, such as culturally diverse counsellors or alternative forms of support, to better meet the needs of international students.

“In the beginning, they assigned different counsellors to us. I think it would be better if we could choose ourselves, for example, by reading counsellor profiles on the school website.” (Participant 06)

“I wish the waiting time could be shorter. Waiting for a month when you’re in pain is unbearable.” (Participant 03)

“I think the main thing is to increase publicity. Many people don’t know about this service, only after I told them did they find out.” (Participant 09)

“It’s impossible for the university to provide counsellors who speak the native language of every international student. But I think the university could, for example, recruit some volunteers to assist. Even if they are not qualified counsellors, they could help with translation during the process. That way, students from countries with less commonly spoken languages could express themselves more clearly and accurately.” (Participant 01)
